# The genome sequence of the Meadow Fritillary, *Melitaea parthenoides* Keferstein, 1851 (Lepidoptera: Nymphalidae)

**DOI:** 10.12688/wellcomeopenres.24718.1

**Published:** 2025-08-11

**Authors:** Yannick Chittaro, Kay Lucek, Charlotte J. Wright, Joana I. Meier, Mark L. Blaxter, Wellcome Sanger Institute Tree of Life Management, Samples and Laboratory team, Wellcome Sanger Institute Scientific Operations: Sequencing Operations, Wellcome Sanger Institute Tree of Life Core Informatics team, Tree of Life Core Informatics collective, Project Psyche Community

**Affiliations:** 1 Info fauna, Neuchâtel, Switzerland; 2 University of Neuchâtel, Neuchâtel, Switzerland; 3 Tree of Life, Wellcome Sanger Institute, Hinxton, England, UK

**Keywords:** Melitaea parthenoides, Meadow Fritillary, genome sequence, chromosomal, Lepidoptera

## Abstract

We present a genome assembly from a female specimen of *Melitaea parthenoides* (Meadow Fritillary; Arthropoda; Insecta; Lepidoptera; Nymphalidae). The assembly contains two haplotypes with total lengths of 592.15 megabases and 568.40 megabases. Most of haplotype 1 (99.72%) is scaffolded into 31 chromosomal pseudomolecules, including the Z sex chromosome. Haplotype 2 was assembled to scaffold level. The mitochondrial genome has also been assembled, with a length of 15.17 kilobases.

## Species taxonomy

Eukaryota; Opisthokonta; Metazoa; Eumetazoa; Bilateria; Protostomia; Ecdysozoa; Panarthropoda; Arthropoda; Mandibulata; Pancrustacea; Hexapoda; Insecta; Dicondylia; Pterygota; Neoptera; Endopterygota; Amphiesmenoptera; Lepidoptera; Glossata; Neolepidoptera; Heteroneura; Ditrysia; Obtectomera; Papilionoidea; Nymphalidae; Nymphalinae; Melitaeini; Melitaeina; *Melitaea*; *Mellicta*; *Melitaea parthenoides* Keferstein, 1851 (NCBI:txid113340)

## Background

The Meadow Fritillary *Melitaea parthenoides* is endemic to Western Europe ( Kudrna *et al.*, 2015). Its range includes the mountainous regions of the Iberian Peninsula ( Gómez Bustillo & Fernández-Rubio, 1974), a large part of France ( Lafranchis *et al.*, 2015), some parts of the western Italian Alps ( Vila *et al.*, 2009), western and northern Switzerland ( Ligue Suisse pour la Protection de la Nature, 1987), and southern Germany ( Reinhardt *et al.*, 2020). A former presence in Austria, in northern Tyrol, seems credible ( Huemer, 2013).

*Meliteae parthenoides* is a medium-sized Fritillary with a relatively regular black pattern on the upper surface of its wings. It closely resembles several other species in the genus, notably *M. athalia*, *M. celadussa* and *M. varia*. The male genitalia are the most reliable distinguishing feature: The tips of the valves are slightly bent downwards, usually with small ventral spines ( van Oorschot & Coutsis, 2014). In females, the background colour is often less uniform, where postdiscal areas are darker and more contrasting.

*Melitaea parthenoides* colonises dry limestone grasslands and extensive mesophilous meadows, as well as wet meadows, especially on the edges of swamps, from the plains to over 2 400 m in some cases ( Gómez Bustillo & Fernández-Rubio, 1974; Lafranchis *et al.*, 2015).

The eggs are laid in patches under the undersides of the leaves of host plants. The young caterpillars live together in a woven nest. Second generation caterpillars overwinter there after the second moult ( Ligue Suisse pour la Protection de la Nature, 1987). The host plants are plantain ( *Plantago lanceolata*, *P. media*), but the caterpillars sometimes eat other plants, especially after overwintering ( *Veronica* spp., *Rhinanthus* spp., *Melampyrum* spp., Reinhardt *et al.*, 2020).

The species is bivoltine at low altitudes (May to June, then August to September) and in warmer locations, whereas at high altitudes or in humid locations it generally forms only one generation in June to July ( Ebert & Rennwald, 1991; Gómez Bustillo & Fernández-Rubio, 1974; Lafranchis *et al.*, 2015; Ligue Suisse pour la Protection de la Nature, 1987).

Although it is still fairly widespread in the south and west of its range it is threatened by the intensification of agricultural practices, such as eutrophication due to the regular application of synthetic fertilisers or manure, and the increase in the number of annual cuttings, as well as overgrazing. This has led to its decline in several regions, and the species is considered threatened in some regions (for example in northern France, Lafranchis *et al.*, 2015) or even countries, such as Germany ( Reinhardt & Bolz, 2011) and Switzerland ( Wermeille *et al.*, 2014).

**Figure 1.**
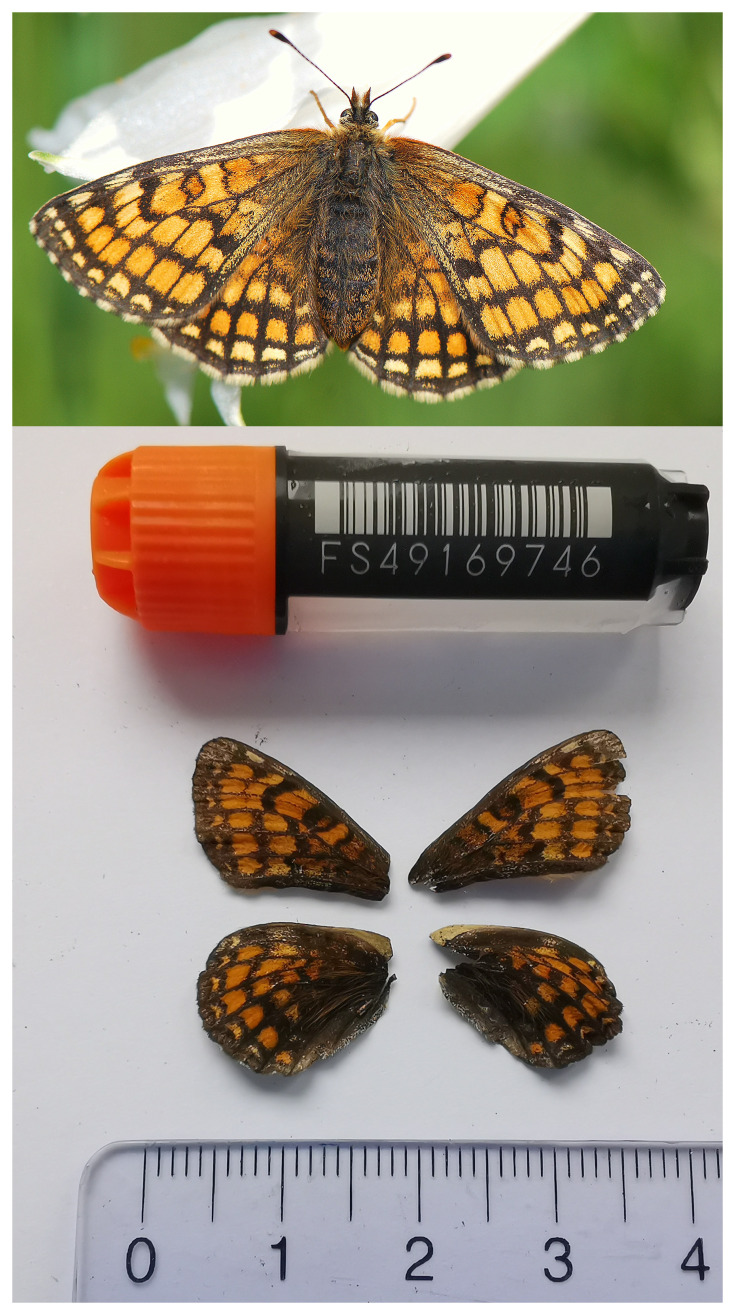
Top: Live specimen of *Melitaea parthenoides*; Bottom: Voucher image of the *Melitaea parthenoides* (ilMelPart1) specimen used for genome sequencing.

This reference genome presented here will help to unravel the complex genetic structure revealed by mitochondrial DNA ( Dapporto *et al.*, 2022). It will also help to conduct future conservation genomic research, especially in the threatened part of the species range. The sequence data was derived from a female specimen ( Figure 1) collected from Dittingen BL, Switzerland.

## Methods

### Sample acquisition

The specimen used for genome sequencing was an adult female *Melitaea parthenoides* (specimen ID SAN28000138, ToLID ilMelPart1; Figure 1), collected from Dittingen BL, Switzerland (latitude 47.4431, longitude 7.4918; elevation 500 m) on 03/06/2023. The specimen was collected and identified by Yannick Chittaro (Info Fauna, Neuchâtel, Switzerland).

### Nucleic acid extraction

Protocols for high molecular weight (HMW) DNA extraction developed at the Wellcome Sanger Institute (WSI) Tree of Life Core Laboratory are available on protocols.io ( Howard *et al.*, 2025). The ilMelPart1 sample was weighed and triaged to determine the appropriate extraction protocol. Tissue from the thorax was homogenised by powermashing using a PowerMasher II tissue disruptor. HMW DNA was extracted in the WSI Scientific Operations core using the Automated MagAttract v2 protocol. DNA was sheared into an average fragment size of 12–20 kb following the Megaruptor®3 for LI PacBio protocol. Sheared DNA was purified by automated SPRI (solid-phase reversible immobilisation). The concentration of the sheared and purified DNA was assessed using a Nanodrop spectrophotometer and Qubit Fluorometer using the Qubit dsDNA High Sensitivity Assay kit. Fragment size distribution was evaluated by running the sample on the FemtoPulse system. For this sample, the final post-shearing DNA had a Qubit concentration of 50.77 ng/μL and a yield of 2 386.19 ng, with a fragment size of 17.0 kb. The 260/280 spectrophotometric ratio was 1.88, and the 260/230 ratio was 2.43.

### PacBio HiFi library preparation and sequencing

Library preparation and sequencing were performed at the WSI Scientific Operations core. Libraries were prepared using the SMRTbell Prep Kit 3.0 (Pacific Biosciences, California, USA), following the manufacturer’s instructions. The kit includes reagents for end repair/A-tailing, adapter ligation, post-ligation SMRTbell bead clean-up, and nuclease treatment. Size selection and clean-up were performed using diluted AMPure PB beads (Pacific Biosciences). DNA concentration was quantified using a Qubit Fluorometer v4.0 (ThermoFisher Scientific) and the Qubit 1X dsDNA HS assay kit. Final library fragment size was assessed with the Agilent Femto Pulse Automated Pulsed Field CE Instrument (Agilent Technologies) using the gDNA 55 kb BAC analysis kit.

The sample was sequenced on a Revio instrument (Pacific Biosciences). The prepared library was normalised to 2 nM, and 15 μL was used for making complexes. Primers were annealed and polymerases bound to generate circularised complexes, following the manufacturer’s instructions. Complexes were purified using 1.2X SMRTbell beads, then diluted to the Revio loading concentration (200–300 pM) and spiked with a Revio sequencing internal control. The sample was sequenced on a Revio 25M SMRT cell. The SMRT Link software (Pacific Biosciences), a web-based workflow manager, was used to configure and monitor the run and to carry out primary and secondary data analysis.

Specimen details, sequencing platforms, and data yields are summarised in Table 1.

**Table 1.** Specimen and sequencing data for BioProject.

Platform	PacBio HiFi	Hi-C
**ToLID**	ilMelPart1	ilMelPart1
**Specimen ID**	SAN28000138	SAN28000138
**BioSample (source individual)**	SAMEA115110004	SAMEA115110004
**BioSample (tissue)**	SAMEA115110026	SAMEA115110025
**Tissue**	thorax	head
**Sequencing platform and model**	Revio	Illumina NovaSeq X
**Run accessions**	ERR13605496	ERR13602159
**Read count total**	1.40 million	727.80 million
**Base count total**	15.66 Gb	109.90 Gb

### Hi-C

***Sample preparation and crosslinking***

The Hi-C sample was prepared from 20–50 mg of frozen head tissue of the ilMelPart1 sample using the Arima-HiC v2 kit (Arima Genomics). Following the manufacturer’s instructions, tissue was fixed and DNA crosslinked using TC buffer to a final formaldehyde concentration of 2%. The tissue was homogenised using the Diagnocine Power Masher-II. Crosslinked DNA was digested with a restriction enzyme master mix, biotinylated, and ligated. Clean-up was performed with SPRISelect beads before library preparation. DNA concentration was measured with the Qubit Fluorometer (Thermo Fisher Scientific) and Qubit HS Assay Kit. The biotinylation percentage was estimated using the Arima-HiC v2 QC beads.

***Hi-C library preparation and sequencing***

Biotinylated DNA constructs were fragmented using a Covaris E220 sonicator and size selected to 400–600 bp using SPRISelect beads. DNA was enriched with Arima-HiC v2 kit Enrichment beads. End repair, A-tailing, and adapter ligation were carried out with the NEBNext Ultra II DNA Library Prep Kit (New England Biolabs), following a modified protocol where library preparation occurs while DNA remains bound to the Enrichment beads. Library amplification was performed using KAPA HiFi HotStart mix and a custom Unique Dual Index (UDI) barcode set (Integrated DNA Technologies). Depending on sample concentration and biotinylation percentage determined at the crosslinking stage, libraries were amplified with 10–16 PCR cycles. Post-PCR clean-up was performed with SPRISelect beads. Libraries were quantified using the AccuClear Ultra High Sensitivity dsDNA Standards Assay Kit (Biotium) and a FLUOstar Omega plate reader (BMG Labtech).

Prior to sequencing, libraries were normalised to 10 ng/μL. Normalised libraries were quantified again and equimolar and/or weighted 2.8 nM pools. Pool concentrations were checked using the Agilent 4200 TapeStation (Agilent) with High Sensitivity D500 reagents before sequencing. Sequencing was performed using paired-end 150 bp reads on the Illumina NovaSeq X.

Specimen details, sequencing platforms, and data yields are summarised in Table 1.

### Genome assembly

Prior to assembly of the PacBio HiFi reads, a database of *k*-mer counts ( *k* = 31) was generated from the filtered reads using FastK. GenomeScope2 ( Ranallo-Benavidez *et al.*, 2020) was used to analyse the *k*-mer frequency distributions, providing estimates of genome size, heterozygosity, and repeat content.

The HiFi reads were assembled using Hifiasm in Hi-C phasing mode ( Cheng *et al.*, 2021; Cheng *et al.*, 2022), producing two haplotypes. Hi-C reads ( Rao *et al.*, 2014) were mapped to the primary contigs using bwa-mem2 ( Vasimuddin *et al.*, 2019). Contigs were further scaffolded with Hi-C data in YaHS ( Zhou *et al.*, 2023), using the --break option for handling potential misassemblies. The scaffolded assemblies were evaluated using Gfastats ( Formenti *et al.*, 2022), BUSCO ( Manni *et al.*, 2021) and MERQURY.FK ( Rhie *et al.*, 2020).

The mitochondrial genome was assembled using MitoHiFi ( Uliano-Silva *et al.*, 2023), which runs MitoFinder ( Allio *et al.*, 2020) and uses these annotations to select the final mitochondrial contig and to ensure the general quality of the sequence.

### Assembly curation

The assembly was decontaminated using the Assembly Screen for Cobionts and Contaminants ( ASCC) pipeline. TreeVal was used to generate the flat files and maps for use in curation. Manual curation was conducted primarily in PretextView and HiGlass ( Kerpedjiev *et al.*, 2018). Scaffolds were visually inspected and corrected as described by Howe *et al*. (2021). Manual corrections included 7 breaks and 20 joins. The curation process is documented at https://gitlab.com/wtsi-grit/rapid-curation. PretextSnapshot was used to generate a Hi-C contact map of the final assembly.

### Assembly quality assessment

Chromosomal painting was performed using lep_busco_painter using Merian elements, which represent the 32 ancestral linkage groups in Lepidoptera ( Wright *et al.*, 2024). Painting was based on gene locations from the lepidoptera_odb10 BUSCO analysis and chromosome lengths from the genome index produced using SAMtools faidx ( Danecek *et al.*, 2021). Each complete BUSCO (including both single-copy and duplicated BUSCOs) was assigned to a Merian element using a reference database, and coloured positions were plotted along chromosomes drawn to scale.

The Merqury.FK tool ( Rhie *et al.*, 2020), run in a Singularity container ( Kurtzer *et al.*, 2017), was used to evaluate *k*-mer completeness and assembly quality for both haplotypes using the *k*-mer databases ( *k* = 31) computed prior to genome assembly. The analysis outputs included assembly QV scores and completeness statistics.

The genome was analysed using the BlobToolKit pipeline, a Nextflow implementation of the earlier Snakemake BlobToolKit pipeline ( Challis *et al.*, 2020). The pipeline aligns PacBio reads using minimap2 ( Li, 2018) and SAMtools ( Danecek *et al.*, 2021) to generate coverage tracks. Simultaneously, it queries the GoaT database ( Challis *et al.*, 2023) to identify relevant BUSCO lineages and runs BUSCO ( Manni *et al.*, 2021). For the three domain-level BUSCO lineages, BUSCO genes are aligned to the UniProt Reference Proteomes database ( Bateman *et al.*, 2023) using DIAMOND blastp ( Buchfink *et al.*, 2021). The genome is divided into chunks based on the density of BUSCO genes from the closest taxonomic lineage, and each chunk is aligned to the UniProt Reference Proteomes database with DIAMOND blastx. Sequences without hits are chunked using seqtk and aligned to the NT database with blastn ( Altschul *et al.*, 1990). The BlobToolKit suite consolidates all outputs into a blobdir for visualisation. The BlobToolKit pipeline was developed using nf-core tooling ( Ewels *et al.*, 2020) and MultiQC ( Ewels *et al.*, 2016), with package management via Conda and Bioconda ( Grüning *et al.*, 2018), and containerisation through Docker ( Merkel, 2014) and Singularity ( Kurtzer *et al.*, 2017).

## Genome sequence report

### Sequence data

The genome of a specimen of *Melitaea parthenoides* was sequenced using Pacific Biosciences single-molecule HiFi long reads, generating 15.66 Gb (gigabases) from 1.40 million reads, which were used to assemble the genome. GenomeScope2.0 analysis estimated the haploid genome size at 573.87 Mb, with a heterozygosity of 1.19% and repeat content of 39.00%. These estimates guided expectations for the assembly. Based on the estimated genome size, the sequencing data provided approximately 26× coverage. Hi-C sequencing produced 109.90 Gb from 727.80 million reads, which were used to scaffold the assembly. Table 1 summarises the specimen and sequencing details.

### Assembly statistics

The genome was assembled into two haplotypes using Hi-C phasing. Haplotype 1 was curated to chromosome level, while haplotype 2 was assembled to scaffold level. The final assembly has a total length of 592.15 Mb in 69 scaffolds, with 116 gaps, and a scaffold N50 of 20.37 Mb ( Table 2).

**Table 2.** Genome assembly statistics.

**Assembly name**	ilMelPart1.hap1.1	ilMelPart1.hap2.1
**Assembly accession**	GCA_964261285.1	GCA_964261255.1
**Assembly level**	chromosome	scaffold
**Span (Mb)**	592.15	568.40
**Number of chromosomes**	31	N/A
**Number of contigs**	185	212
**Contig N50**	6.74 Mb	6.24 Mb
**Number of scaffolds**	69	98
**Scaffold N50**	20.37 Mb	20.13 Mb
**Longest scaffold length (Mb)**	27.92	N/A
**Sex chromosomes**	Z	N/A
**Organelles**	Mitochondrial genome: 15.17 kb	N/A

**Figure 2.**
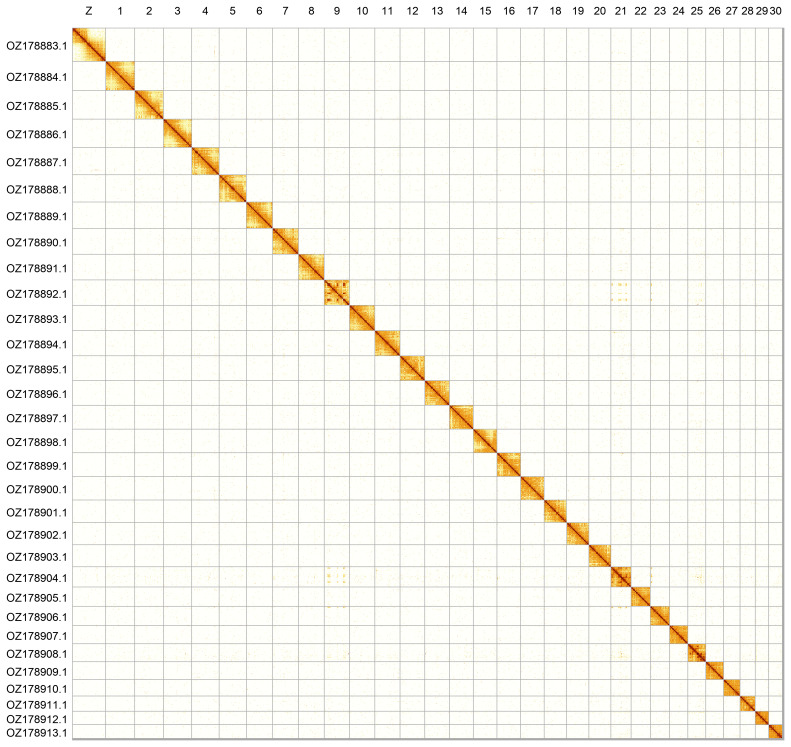
Hi-C contact map of the *Melitaea parthenoides* genome assembly. Assembled chromosomes are shown in order of size and labelled along the axes. The plot was generated using PretextSnapshot.

**Figure 3.**
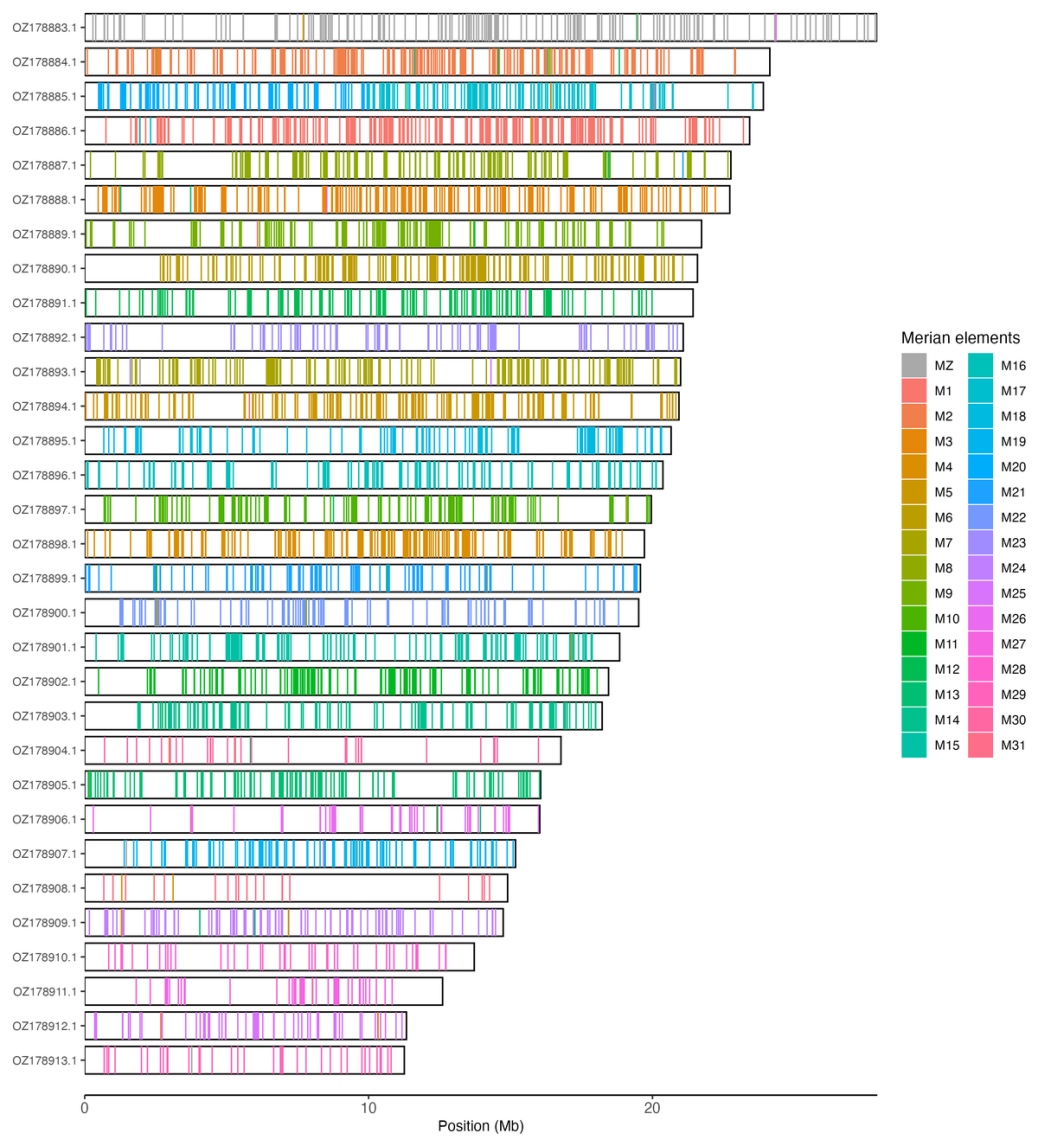
Merian elements painted across chromosomes in the ilMelPart1.hap1.1 assembly of *Melitaea parthenoides*. Chromosomes are drawn to scale, with the positions of orthologues shown as coloured bars. Each orthologue is coloured by the Merian element that it belongs to. All orthologues which could be assigned to Merian elements are shown.

Most of the assembly sequence (99.72%) was assigned to 31 chromosomal-level scaffolds, representing 30 autosomes and the Z sex chromosome. Chromosome Z was assigned based on Hi-C signal. The species appears to be ZO. The chromosome-level scaffolds, confirmed by Hi-C data, are named according to size ( Figure 2; Table 3). Chromosome painting with Merian elements illustrates the distribution of orthologues along chromosomes and highlights patterns of chromosomal evolution relative to Lepidopteran ancestral linkage groups ( Figure 3).

**Table 3.** Chromosomal pseudomolecules in the haplotype 1 genome assembly of *Melitaea parthenoides* ilMelPart1.

INSDC accession	Molecule	Length (Mb)	GC%	Assigned Merian elements
OZ178884.1	1	24.13	34	M2
OZ178885.1	2	23.90	34.50	M17;M20
OZ178886.1	3	23.42	34.50	M1
OZ178887.1	4	22.76	34	M8
OZ178888.1	5	22.72	34.50	M3
OZ178889.1	6	21.73	34	M9
OZ178890.1	7	21.58	34	M6
OZ178891.1	8	21.43	34	M12
OZ178892.1	9	21.08	34.50	M23
OZ178893.1	10	20.99	34.50	M7
OZ178894.1	11	20.93	34	M5
OZ178895.1	12	20.66	34	M18
OZ178896.1	13	20.37	34	M16
OZ178897.1	14	19.96	34.50	M10
OZ178898.1	15	19.71	34	M4
OZ178899.1	16	19.57	34	M21
OZ178900.1	17	19.51	34	M22
OZ178901.1	18	18.84	34	M15
OZ178902.1	19	18.45	34.50	M11
OZ178903.1	20	18.23	35	M14
OZ178904.1	21	16.78	36.50	M30
OZ178905.1	22	16.06	34	M13
OZ178906.1	23	16.03	34.50	M26
OZ178907.1	24	15.18	35	M19
OZ178908.1	25	14.90	37	M31
OZ178909.1	26	14.74	34.50	M24
OZ178910.1	27	13.72	34.50	M28
OZ178911.1	28	12.61	34.50	M27
OZ178912.1	29	11.33	35	M25
OZ178913.1	30	11.26	35.50	M29
OZ178883.1	Z	27.92	34	MZ

The mitochondrial genome was also assembled. This sequence is included as a contig in the multifasta file of the genome submission and as a standalone record.

### Assembly quality metrics

**Figure 4.**
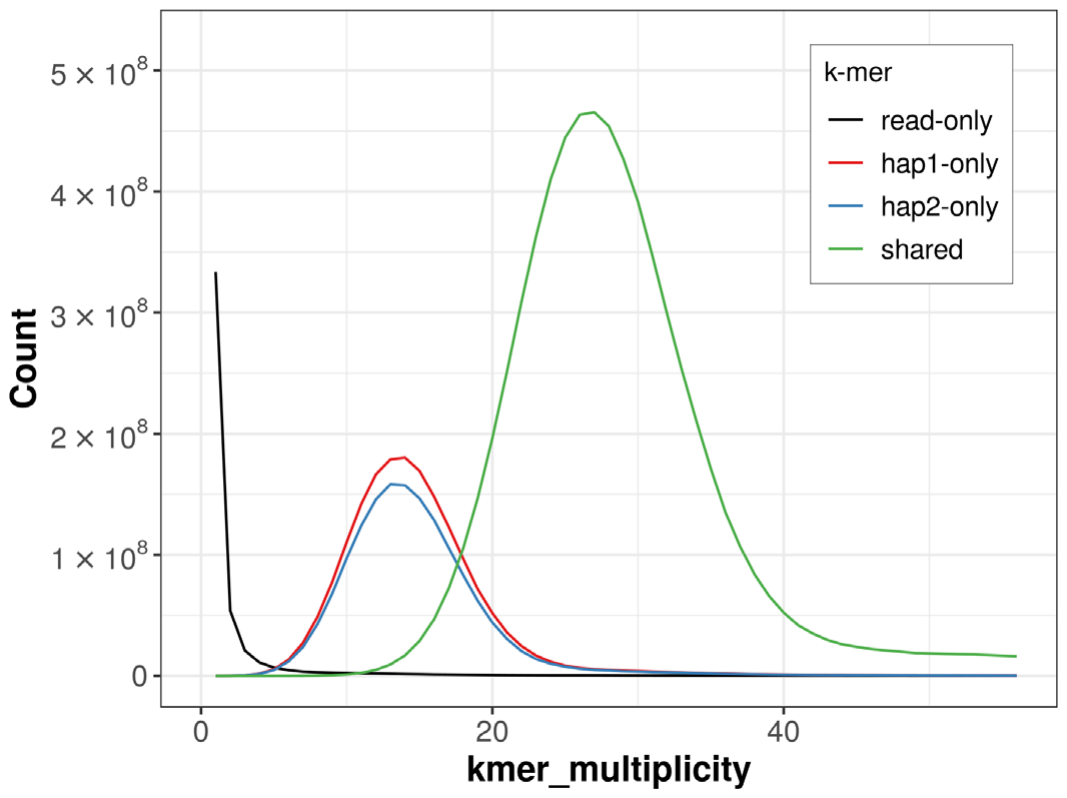
Evaluation of *k*-mer completeness using MerquryFK. This plot illustrates the recovery of *k*-mers from the original read data in the final assemblies. The horizontal axis represents *k*-mer multiplicity, and the vertical axis shows the number of *k*-mers. The black curve represents *k*-mers that appear in the reads but are not assembled. The green curve (the homozygous peak) corresponds to *k*-mers shared by both haplotypes and the red and blue curves (the heterozygous peaks) show *k*-mers found only in one of the haplotypes.

**Figure 5.**
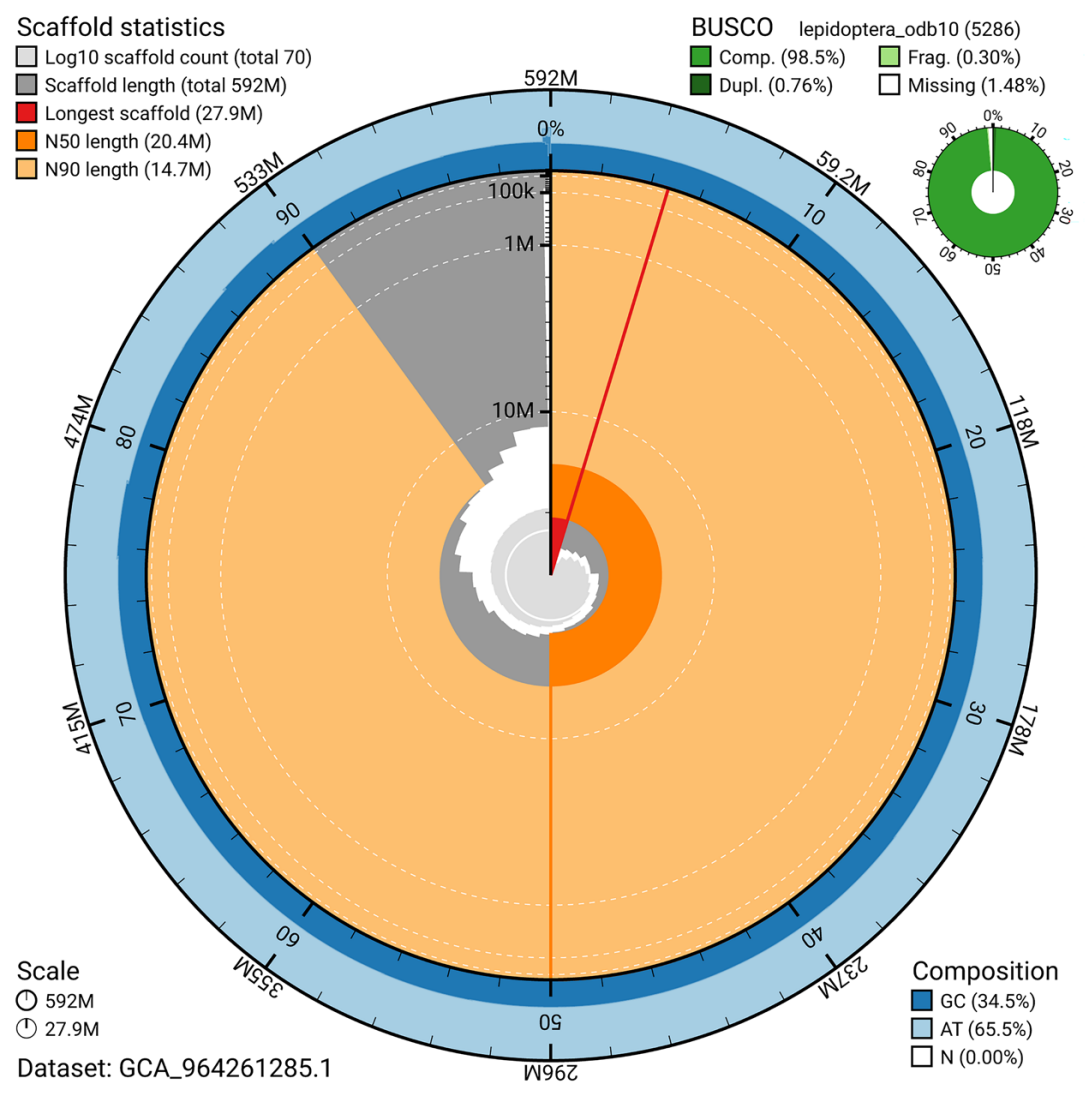
Assembly metrics for ilMelPart1.hap1.1. The BlobToolKit snail plot provides an overview of assembly metrics and BUSCO gene completeness. The circumference represents the length of the whole genome sequence, and the main plot is divided into 1,000 bins around the circumference. The outermost blue tracks display the distribution of GC, AT, and N percentages across the bins. Scaffolds are arranged clockwise from longest to shortest and are depicted in dark grey. The longest scaffold is indicated by the red arc, and the deeper orange and pale orange arcs represent the N50 and N90 lengths. A light grey spiral at the centre shows the cumulative scaffold count on a logarithmic scale. A summary of complete, fragmented, duplicated, and missing BUSCO genes in the set is presented at the top right. An interactive version of this figure can be accessed on the BlobToolKit viewer.

**Figure 6.**
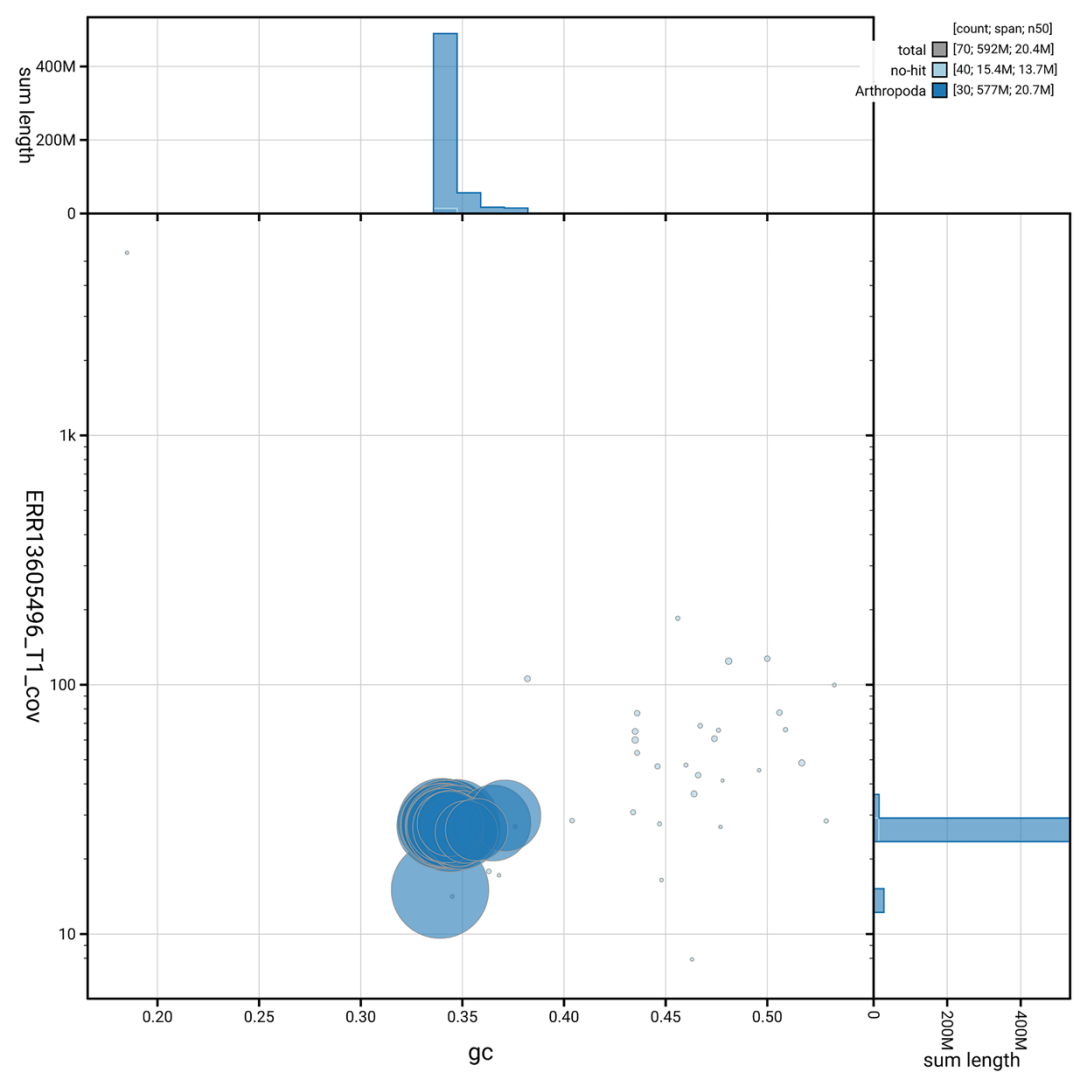
BlobToolKit GC-coverage plot for ilMelPart1.hap1.1. Blob plot showing sequence coverage (vertical axis) and GC content (horizontal axis). The circles represent scaffolds, with the size proportional to scaffold length and the colour representing phylum membership. The histograms along the axes display the total length of sequences distributed across different levels of coverage and GC content. An interactive version of this figure is available on the BlobToolKit viewer.

For haplotype 1, the estimated QV is 63.7, and for haplotype 2, 63.7. When the two haplotypes are combined, the assembly achieves an estimated QV of 63.7. The *k*-mer completeness is 77.06% for haplotype 1, 73.81% for haplotype 2, and 99.31% for the combined haplotypes ( Figure 4). BUSCO analysis using the lepidoptera_odb10 reference set ( *n* = 5 286) ( Kriventseva *et al.*, 2019) identified 98.5% of the expected gene set (single = 97.8%, duplicated = 0.8%) for haplotype 1. The snail plot in Figure 5 summarises the scaffold length distribution and other assembly statistics for haplotype 1. The blob plot in Figure 6 shows the distribution of scaffolds by GC proportion and coverage for haplotype 1.

Table 4 lists the assembly metric benchmarks adapted from Rhie *et al.* (2021) the Earth BioGenome Project Report on Assembly Standards September 2024. The EBP metric, calculated for the haplotype 1, is **6.C.Q63**, meeting the recommended reference standard.

**Table 4.** Earth Biogenome Project summary metrics for the *Melitaea parthenoides* assembly.

Measure	Value	Benchmark
EBP summary (haplotype 1)	6.7.Q63	6.C.Q40
Contig N50 length	6.74 Mb	≥ 1 Mb
Scaffold N50 length	20.37 Mb	= chromosome N50
Consensus quality (QV)	Haplotype 1: 63.7; haplotype 2: 63.7; combined: 63.7	≥ 40
*k*-mer completeness	Haplotype 1: 77.06%; Haplotype 2: 73.81%; combined: 99.31%	≥ 95%
BUSCO	C:98.5%[S:97.8%‚D:0.8%]‚ F:0.3%‚M:1.2%‚n:5 286	S > 90%; D < 5%
Percentage of assembly assigned to chromosomes	99.72%	≥ 90%

### Wellcome Sanger Institute – Legal and Governance

The materials that have contributed to this genome note have been supplied by a Tree of Life collaborator. The Wellcome Sanger Institute employs a process whereby due diligence is carried out proportionate to the nature of the materials themselves, and the circumstances under which they have been/are to be collected and provided for use. The purpose of this is to address and mitigate any potential legal and/or ethical implications of receipt and use of the materials as part of the research project, and to ensure that in doing so, we align with best practice wherever possible. The overarching areas of consideration are:

- Ethical review of provenance and sourcing of the material
- Legality of collection, transfer and use (national and international).

Each transfer of samples is undertaken according to a Research Collaboration Agreement or Material Transfer Agreement entered into by the Tree of Life collaborator, Genome Research Limited (operating as the Wellcome Sanger Institute), and in some circumstances, other Tree of Life collaborators.

## Data Availability

European Nucleotide Archive: Mellicta parthenoides (meadow fritillary). Accession number PRJEB79166. The genome sequence is released openly for reuse. The *Melitaea parthenoides* genome sequencing initiative is part of the Sanger Institute Tree of Life Programme (PRJEB43745) and Project Psyche (PRJEB71705). All raw sequence data and the assembly have been deposited in INSDC databases. The genome will be annotated using available RNA-Seq data and presented through Ensembl at the European Bioinformatics Institute. Raw data and assembly accession identifiers are reported in Table 1 and Table 2. Pipelines used for genome assembly at the WSI Tree of Life are available at https://pipelines.tol.sanger.ac.uk/pipelines. Table 5 lists software versions used in this study.

## Author information

Contributors are listed at the following links:

- Wellcome Sanger Institute Tree of Life Management, Samples and Laboratory team
- Wellcome Sanger Institute Scientific Operations – Sequencing Operations
- Wellcome Sanger Institute Tree of Life Core Informatics team
- Tree of Life Core Informatics collective
- Project Psyche Community.
