# Supplementary material for: The genome sequence of the Meadow Fritillary, Melitaea parthenoides Keferstein, 1851 (Lepidoptera: Nymphalidae)
Source: Wellcome Open Res. 2025 Aug 11;10:436. [Version 1] doi: 10.12688/wellcomeopenres.24718.1 (PMC12579322; doi:10.12688/wellcomeopenres.24718.1)
Supplement: Peer review history

# Reviewer report: Reviewer response for version 1

**DOI:** 10.21956/wellcomeopenres.27234.r134033

**Published:** 2025-10-31

**Authors:** Arun Arumugaperumal

**Affiliations:** 1 Department of Biotechnology, Rajalakshmi Engineering College, Thandalam 602105, Chennai, Tamilnadu, India

The authors have presented the genome sequence of *Melitaea parthenoides*, also known as the Meadow Fritillary. The assembly reported is of size 592.15 Mb and the mitogenome is of size 15.17 kb. The authors have sequenced a female specimen, and they have captured the Z chromosome. They have commented that this butterfly follows the ZO sex system. It is interesting to note that some members of the same genus have ZW sex system [(Chittaro Y, et al., 2025) - ref 1] and others have ZO system [(Wymann H, et al., 2025) - ref 2] [(Chittaro Y, et al., 2025) - ref 3]. The photograph provided in Figure 1 will be very helpful to the readers. The sequencing procedure has been performed according to the standard tree of life project protocols. A high-quality genome sequence has been reported as evident from N50 values. BUSCO completeness value of 98.5% indicates that the genome is a near-complete one with respect to conserved gene sequences present in the database. The GC blob plot also shows that there is no contaminating sequence from any other phylum.

 The EBP metric has been mentioned as 6.C.Q63 in the text. But in Table 4, it is mentioned as 6.7.Q63. Kindly correct this discrepancy. After the correction, the article can be indexed.

Are sufficient details of methods and materials provided to allow replication by others?

Yes

Is the rationale for creating the dataset(s) clearly described?

Yes

Are the datasets clearly presented in a useable and accessible format?

Yes

Are the protocols appropriate and is the work technically sound?

Yes

Reviewer Expertise:

Bioinformatics; Genomics

I confirm that I have read this submission and believe that I have an appropriate level of expertise to confirm that it is of an acceptable scientific standard.

## References

[1] : The genome sequence of the Grisons Fritillary, Melitaea varia Herrich-Schaeffer, 1851 (Lepidoptera: Nymphalidae). *Wellcome Open Research* .2025;10: 10.12688/wellcomeopenres.24686.1 10.12688/wellcomeopenres.24686.1 39944230

[2] : The genome sequence of the False Heath Fritillary, Melitaea diamina (Lang, 1789) (Lepidoptera: Nymphalidae). *Wellcome Open Research* .2025;10: 10.12688/wellcomeopenres.24719.1 10.12688/wellcomeopenres.24719.1 41103340 PMC12521902

[3] : The genome sequence of the Nickerl’s Fritillary, Melitaea aurelia Nickerl, 1850 (Lepidoptera: Nymphalidae). *Wellcome Open Research* .2025;10: 10.12688/wellcomeopenres.24749.1 10.12688/wellcomeopenres.24749.1 41050512 PMC12495394

# Reviewer report: Reviewer response for version 1

**DOI:** 10.21956/wellcomeopenres.27234.r130207

**Published:** 2025-09-10

**Authors:** Evan J Kipp

**Affiliations:** 1 University of Minnesota Twin Cities Institute on the Environment, Saint Paul, Minnesota, USA

In this data note, Chittaro and colleagues describe the sequencing and assembly of the genome of *Melitaea parthenoides *(Lepidoptera: Nymphalidae), the European Meadow Fritillary. This effort represents the first genome assembly for this species and is one of a growing number of published Fritillary genomes. This high-quality genome resource will contribute to future comparative genomic and evolutionary studies across Lepidoptera and other insects. 

 Overall, this report is clearly written, and the methods are well described. The authors provide valuable background on the natural history of this species and highlight some threats that have led to recent population declines over certain portions of its range. Using a combination of HiFi reads and Hi-C short-reads, two haplotype assemblies were produced, both with relatively high contiguity. The primary haplotype was used to generate 31 chromosome-level scaffolds, which included the Z sex chromosome and 30 autosomes. All detailed assessments of quality metrics, including BUSCO analysis, indicate that this genome assembly is of high quality and meets the standards outlined by the Earth Biogenome Project. 

 Minor points: 

 The authors report k-mer completeness findings for both haplotypes 1 and 2, however, BUSCO analysis was only reported for the primary haplotype. Was haplotype 2 also assessed using BUSCO against the lepidoptera_odb10 database and, if so, what were the resulting values?

 I do think that some additional details are warranted surrounding the description of Merian elements in this genome. In the Methods, the authors note that each complete BUSCO was assigned to an ancestral Merian element using a 'reference database' but do not detail how this was performed or what database was used. If this was performed using publicly available tools and databases, these should be included. Additionally, for myself and other readers who may not be deeply familiar with lepidopteran genomics, it would be helpful to include an additional sentence or two describing these findings. Are the Merian element patterns presented in Figure 3 and Table 3 consistent with other Fritillary genomes or are there any noteworthy differences that may be worthy of future investigation?

Are sufficient details of methods and materials provided to allow replication by others?

Yes

Is the rationale for creating the dataset(s) clearly described?

Yes

Are the datasets clearly presented in a useable and accessible format?

Yes

Are the protocols appropriate and is the work technically sound?

Yes

Reviewer Expertise:

phylogenetics, genomics

I confirm that I have read this submission and believe that I have an appropriate level of expertise to confirm that it is of an acceptable scientific standard.
